# Organic particle scavenging by marine bacteria: influences of bacterial nanoscale surface properties

**DOI:** 10.1128/aem.01049-25

**Published:** 2025-06-16

**Authors:** Yosuke Yamada, Toshiaki Mochizuki, Nirav Patel, Farooq Azam, Hideki Fukuda, Toshi Nagata, Satoshi Mitarai

**Affiliations:** 1 Kochi Institute for Core Sample Research, Institute for Extra-cutting-edge Science and Technology Avant-garde Research, Japan Agency for Marine-Earth Science and Technology (JAMSTEC) 57919 , Nankoku, Kochi, Japan; 2 Advanced Institute for Marine Ecosystem Change (WPI-AIMEC), JAMSTEC 13570 , Yokohama, Kanagawa, Japan; 3 Okinawa Institute of Science and Technology Graduate University 56874, Onna, Okinawa, Japan; 4 Scripps Institution of Oceanography 70015 , La Jolla, California, USA; 5 Atmosphere and Ocean Research Institute, The University of Tokyo 74024 , Kashiwa, Chiba, Japan; 6 Super-cutting-edge Grand and Advanced Research (SUGAR) Program, Institute for Extra-cutting-edge Science and Technology Avant-garde Research (X-star), Japan Agency for Marine-Earth Science and Technology (JAMSTEC) 13570, Yokosuka, Kanagawa, Japan; University of Delaware, Lewes, Delaware, USA

**Keywords:** bacterial surface, Young’s modulus, adhesiveness, nanoparticle, marine biogeochemical cycles, marine ecology

## Abstract

Marine bacteria contribute to biogeochemical cycles by scavenging organic nanoparticles such as cell fragments, viruses, excretions from phytoplankton and bacteria, and naturally occurring polymeric substances. Nonetheless, bacterial surface properties influencing this process remain poorly understood. A previous study found that marine bacteria exhibit significant variation in surface roughness, which affects nanoparticle attachment, influencing bacterial survival and marine biogeochemical cycles. However, cell surface characteristics such as Young’s modulus and adhesiveness have rarely been measured. This study investigated bacterial nanoscale surface properties and their effects on nanoparticle attachment. Atomic force microscopy was employed to measure these parameters of 559 individual bacterial cells collected from Okinawa coastal waters. These results revealed significant variation in Young’s modulus (6–21,000 kPa) and adhesiveness (86–1,200 pN). Subsequent coincubation experiments with polystyrene beads and virus-like particles, as model nanoparticles, demonstrated a significant negative correlation between Young’s modulus and the attachment of virus-like particles, whereas no significant relationships were observed for other factors. Our results suggest that measuring bacterial surface properties provides novel insights into their strategies for resource utilization and their contribution to marine biogeochemical cycles.

**IMPORTANCE:**

Surface properties of marine bacteria are believed to influence their ability to acquire nanoparticles for nutrition. However, studies on these properties are limited, and the relationship with nanoparticle attachment remains unclear. This study measured Young’s modulus and adhesiveness of marine bacteria, investigating their variability and their influence upon nanoparticle attachment. This work sheds light on biophysical mechanisms driving bacterial nanoparticle utilization, as well as ecological and biogeochemical implications of bacterial surface properties in marine environments.

## INTRODUCTION

In marine environments, bacterial outer membranes and extracellular structures serve as the main reactive biological surfaces. On these surfaces, various polymers and particles are hydrolyzed by cell surface-bound enzymes, and phytoplankton-derived organic carbon is converted back to its inorganic form (1). Bacteria consume about half of the primary carbon generated in seawater (2). Seawater contains an abundance of organic nanoparticles, or colloidal organic matter, including cell fragments, viruses, excretions from phytoplankton and bacteria, and other naturally occurring polymeric substances (3–5). The utilization of these organic compounds by bacteria, along with the transfer of carbon to higher trophic levels, is referred to as the microbial loop, which recycles primary carbon sources derived from phytoplankton photosynthesis in the ocean (6). Despite the importance of nanoparticles as crucial nutrient sources for bacteria (7–9), bacterial nanoparticle utilization in seawater remains poorly investigated.

In order for bacteria to utilize nanoparticles, the following processes are required: i) an initial encounter, ii) subsequent attachment, iii) enzymatic degradation of particles, and iv) uptake of resulting degradation products (1). Among these processes, nanoparticle scavenging, the initial step in nanoparticle utilization, is influenced by bacterial surface structure, composition, and charge. Furthermore, the encounter probability between bacteria and nanoparticles is governed by their Brownian motion, which is regulated by absolute temperature and viscosity, as described by the Stokes–Einstein model.

Generally, physical forces affecting the attachment of objects and elastic bodies are controlled by Young’s modulus (an indicator of resistance to deformation), adhesiveness, contact area, and Poisson ratio (10, 11). For bacteria, Poisson ratio is commonly assumed to be 0.5 (perfect incompressibility), with the remaining three independent properties considered crucial for attachment. These properties affect the contact area and electrostatic repulsion between nanoparticles and bacterial cells (12–14). Using an atomic force microscope (AFM), Y. Yamada et al. (15) investigated the surface roughness of marine bacteria in coastal and offshore regions in the eastern and western Pacific. Bacterial surface roughness is defined as the standard deviation of height values in a scanned area of a cell and is considered proportional to the contact area between bacteria and nanoparticles. Their findings revealed significant variation in roughness among individual cells, and there was a positive correlation between roughness and the number of scavenged nanoparticles, indicating that bacterial surface roughness substantially affects nanoparticle attachment by modulating the contact surface area. In addition to roughness, however, the relationship between Young’s modulus, adhesiveness, and nanoparticle scavenging remains largely unclear.

Young’s modulus, expressed in pascals (newtons per square meter), defines a material’s stiffness as the ratio of applied stress (force per unit area) to resulting strain (fractional change in length) (16). A higher Young’s modulus indicates greater resistance to deformation, whereas a lower value signifies greater deformability. In bacteria, extensive measurements, particularly on *Escherichia coli* and other pathogens, have reported values ranging from 40 to 769,000 kPa (Table S1). Young’s modulus reflects mechanical properties of the cell envelope and extracellular structures, varying significantly by species and culture conditions. These variations are believed to profoundly impact bacterial physiology and survival (17). Various established theories in contact mechanics have related elastic deformation and particle attachment, suggesting that deformation influences van der Waals and electrostatic forces (10). However, experimental verification remains insufficient, even in the fields of colloid and nanoparticle research (18, 19). To our knowledge, there have been few studies investigating the effects of Young’s modulus on nanoparticle attachment to marine bacteria. Nevertheless, it is recognized that Young’s modulus varies depending on the type of bacteria and the location on the cell surface (20–22). Reports indicate that nanoparticle attachment is influenced by Young’s modulus of cell membranes, including substances and structures on the cell membrane (12, 23, 24), and those of nanoparticles (25).

Bacterial pili and extracellular matrices can enhance or diminish adhesiveness, affecting cell-cell, cell-host, and cell-substrate interactions (26). Although the relationship between adhesiveness and nanoparticle attachment has been reported in various fields, studies focusing on the ocean or on heterogeneous soft, multilayered nanoparticles, such as organic nanoparticles and bacteria, are scarce. This issue is further complicated by the difficulty in applying traditional Derjaguin-Landau-Verwey-Overbeek (DLVO) theory to predict particle attachment because the DLVO theory assumes a uniform surface and evenly distributed charge, an assumption that does not align with the characteristics of marine nanoparticles (27). Only in the case of bacteriophage attachment has it been demonstrated that hydrophobicity and electrostatic interactions of both attaching and attached surfaces, as well as the roughness of the attachment surface, significantly influence adhesion (28–30).

Based on these theories and related research, both Young’s modulus and adhesion could impact nanoparticle attachment to bacteria in seawater. However, the paucity of data regarding these properties of marine bacteria makes it difficult to assess their influence on nanoparticle attachment and their ecological significance.

Therefore, we investigated variation in Young’s modulus and adhesion of bacterial cell surfaces of marine bacterial assemblages and isolates using AFM and determined the relationship between cell surface properties and nanoparticle attachment.

AFM enables direct quantitative assessments and visualization at the nanoscale. In this study, the term “adhesiveness” refers to the adhesive force between the AFM tip and the bacterial cell surface. This adhesive force is assumed to be determined by intrinsic adhesiveness and the effective contact surface area, a definition adapted from the study of fine particle attachment (31). Also, polystyrene beads (PSBs) and virus-like particles (VLPs) were used as model organic nanoparticles. PSBs are spherical, making their attachment easy to observe and to quantify. Furthermore, their surface charge can be controlled through functional groups, making them convenient for investigating attachment mechanisms. Additionally, we define VLPs used in this study as viruses and gene transfer agents (non-virus particles) (32, 33). VLPs are negatively charged nanoparticles (34, 35) commonly found in the ocean, and by staining their nucleic acids with fluorescent dyes, we can quantify their attachment. Using this microscope and model nanoparticles, we demonstrate that Young’s modulus and adhesiveness of marine bacteria exhibit wide taxonomic and individual variations and investigate how these surface properties affect nanoparticle scavenging.

## MATERIALS AND METHODS

### Preparation of natural bacterial assemblages for atomic force microscopy measurements

Seawater samples collected from the coast of Okinawa, Japan (26°26'53.6"N; 127°48'03.5"E) on the 27th and 30th of October and the 6th, 10th, and 13th of November 2020 were filtered through 3.0 µm syringe filters (MCE025300; Membrane Solutions), and 1 mL of filtrate was placed in glass-bottomed dishes (FD35-100, FluoroDish Cell Culture Dish; WPI, UK) coated with poly-D-lysine hydrobromide (final conc. 2  mg mL^−1^; P6407, Sigma-Aldrich). After waiting 30  min for bacterial attachment to dish bottoms at room temperature in the dark, the liquid was removed with an aspirator, and wells were rinsed twice with 1 mL of 0.02-μm-filtered and autoclaved artificial seawater (Daigo’s Artificial Seawater SP for Marine Microalgae Medium; Shiotani M.S., Japan) (<0.02-μm-filtered FASW). Then, dishes were filled with 3 mL of <0.02-μm-filtered FASW. To prevent bacterial surfaces from drying, rinsing was done immediately. Surface properties of attached bacteria on the dish in <0.02-μm-filtered FASW were measured using an AFM within 3‒9 h.

### Preparation of bacterial isolates for AFM and chemical property measurements

Nine gamma proteobacterial taxa randomly isolated from coastal waters and identified to genus or species by 16S rRNA sequence analysis (outsourced to Macrogen Japan Corp. [Tokyo, Japan]; Table S2) were cultured in Marine Broth 2216 medium (Becton, Dickinson, and Company) at room temperature (RT) in the dark for 15‒18 h on a shaker (NR-80; Taitec, Saitama, Japan, 100 rpm). Then, solutions of cultured bacteria were centrifuged (4,000 × *g*, 25 ^o^C, 5 min), and supernatants were discarded. Pellets were suspended in 10 mL of <0.02-μm-filtered FASW and centrifuged again to wash bacteria. Pellets were then resuspended in 1 mL of <0.02-μm-filtered FASW. Washed and concentrated isolates (ca. 10^8–9^ cells mL^−1^) were diluted either x10 or x100 with <0.02-μm-filtered FASW depending on isolate abundance and immediately used for further preparation for AFM (see above) and chemical property measurements (see the supplemental methods).

### AFM measurement of bacterial surface properties

Bacterial image acquisition and measurements of nanoscale surface properties were simultaneously conducted using AFM (QI mode, Nanowizard 4; JPK Instruments, Berlin, Germany). Each measurement area was 5  ×  5 µm with an image resolution of 128  ×  128 pixels; i.e., each pixel was approximately 40  ×  40 nm². To create representative images of bacteria, observations and measurements employed a resolution of 256  ×  256 pixels. That is, each pixel measured approximately 20  ×  20 nm². We used a positively charged silicon nitride probe (BL-AC40TS; Olympus) with a spring constant of 0.1 N m^−1^. The standard contact force (amount of probe indentation) was set to 0.3 nN, and the vertical speed of the probe and the scan rate were set at 100–250 nm ms⁻¹ and 0.3–0.8 Hz, respectively, while observing the shape of the force curve, i.e., ensuring that the probe was indenting the cell and detaching from the cell sufficiently. Surface parameters of each bacterium were calculated from obtained measurements, and images were also generated using the software JPKSPM Data Processing, ver. 6.1.172 (JPK Instruments).

Measurements of bacterial surface properties were obtained by pressing an AFM probe approximately tens of nanometers into the sample and retracting it. Movement was recorded to measure the height and positive and negative changes of forces exerted on the probe to generate a force curve. Measurements of Young’s modulus and adhesiveness were conducted following the manufacturer’s instructions, and mean surface properties for each bacterial cell were calculated from 1 to 5 areas (depending on cell size) of 400  ×  400  nm². Each area included 100 individual force curve measurements, and each measurement was conducted in a 40  ×  40 nm² area near the centers of cells (except for ca. 100  nm distance from cell edges where the values of surface properties could be strongly affected).

Briefly, for Young’s modulus, we determined the baseline of the force curve for each pixel, identified the contact point with the sample, and performed elasticity fitting on the force curve as the probe pressed into the sample surface. That is, the curve generated from changes in positive force applied to the AFM probe when pressed into the sample was fitted with a contact mechanics model to measure Young’s modulus. A rapid increase in positive force indicated a higher Young’s modulus, whereas a gradual increase suggested a lower Young’s modulus (36, 37). This calculation considered the shape of the probe (BL-AC40TS), which was a triangular pyramid, and the half angle to the sample was 19.47 ^o^. The Poisson ratio was 0.5 (a general value for cells), and the Hertz model was used for curve fitting (JPKSPM Data Processing, ver. 6.1.172).

For adhesiveness, we determined the baseline of the force curve for each pixel and calculated the integral of the negative force (the total negative area) as the adhesiveness was overcome during probe retraction from the sample surface (38, 39). In this study, adhesiveness refers to the strength of attachment between the sample surface and the positively charged, silicon nitride AFM probe (40).

### AFM measurement of bacterial cell size

Bacterial cell sizes (equivalent spherical diameters [ESDs]) were calculated from cell volume, treating cells as spheres. We estimated bacterial cell volume by the formula *V* = 4/3 *π*  ×  *a*  ×  b  × *c*, where *a*, *b*, and *c* are semi-axes of a solid ellipse (*a* = *L*/2, *b* = *S*/2, *c* = *H*/2) using AFM-based measurements of *L*, *S*, and *H* from height images (where *L* is the long axis, *S* is the short axis, and *H* is the peak height) (41).

### Epifluorescence microscopy and bacterial-/virus-like particle counts

Bacteria and VLPs were observed and counted using epifluorescence microscopy (42, 43). Briefly, samples fixed with formaldehyde (2% final conc.) were filtered through 0.2 µm polycarbonate (Whatman) for bacteria or 0.02 µm aluminum oxide (Anodisc) filters (Whatman) for VLPs with a vacuum of less than 150  mmHg. Then, they were stained with 4',6-diamidino-2-phenylindole, dihydrochloride (DAPI; final conc. 1.5  µg mL^−1^ in antifade mounting medium; Vectashield; Vector Laboratories) for bacteria or SYBR Green I (Molecular Probes; final conc.: 5.0  ×  10^−3^ of commercial stock in Vectashield) for VLPs for 10  min in the dark and mounted on a glass slide. Slides were observed using epifluorescence microscopy (Nikon Eclipse T*i*; Nikon and Olympus BX51) with 1,000 × magnification for both bacteria and VLPs, and digital images were captured. Abundances of bacteria and VLPs were counted manually on the screen with 20 areas for each sample.

### VLP attachment to bacterial isolates

Viral assemblages were easily detectable due to their 20–200 nm diameters (44). Direct counts of VLP attachment to bacteria (using SYBR Green I) were not feasible using epifluorescence, due to the high fluorescence of bacteria compared to that of VLP. Additionally, measurement using scanning electron microscopy (SEM) proved challenging as distinguishing VLPs from other particles is difficult, and identifying VLPs on the surface of bacteria is likewise challenging. VLP attachment to bacteria was determined using a centrifuge-based method (15, 45). Briefly, 1.5  mL of 0.2-μm-filtered seawater obtained from the coast of Kochi, Japan (33°31'11.6"N 133°45'21.6"E) on 13 Mar 2024 (containing VLP: final conc. 1.0  ×  10^7^ particles mL^−1^) was incubated with bacterial isolates (final conc. 1.0  ×  10^6^ cells mL^−1^) in 1.5 mL polypropylene tubes (bacteria addition treatment). Also, 0.2-μm-filtered seawater without added bacterial isolates was incubated as a control (VLP only control: final conc. 1.0  ×  10^7^ particles mL^−1^). After 3 h incubation at RT in the dark on a shaker (NR-80; Taitec, 100 rpm), samples were centrifuged (4,000 × *g*, 18°C, 5  min), which caused the bacteria to settle to the bottom. We assumed that VLPs attached to bacterial cell surfaces would also settle to the bottom of the tube. Then, 500  µL of supernatant, fixed with formaldehyde (final conc. 2%), was filtered through 0.02 µm Anodisc filters (Whatman) for VLP counting with SYBR Green I staining, followed by epifluorescence microscopy (see above). Abundance of VLPs attached to bacteria (particles mL^−1^) was calculated by subtracting VLP abundance in bacterial treatment samples from that in VLP-only controls. Attached VLP numbers per bacterial cell (particles cell^−1^) were calculated using bacterial abundance after incubation (cells mL^−1^).

### Polystyrene bead attachment to bacterial isolates

PSBs with a diameter of 90–200  nm (mean: 120  nm) (SPHERO Fluorescent Particles, Light Yellow; Spherotech Inc.) were used as model nanoparticles. Prior to incubation, the original PSB solution was diluted 10^5^ times with <0.02-μm-filtered FASW and sonicated for 30  s at ca. 5  kHz (50 Sonic Dismembrator, Fisher Scientific). This PSB suspension was filtered through a 0.01 µm polycarbonate filter (25 mm diameter; GVS) to quantify PSB abundance. Observations using SEM revealed that the abundance of PSB was ca. 10^8^ particles mL^−1^ after 10^5^ dilution and confirmed that most PSBs were not aggregated. After this preparation, the PSB suspension was immediately used for further experiments.

PSBs attached to bacteria were counted with SEM. One mL of < 0.02-μm-filtered FASW containing PSBs (final conc. 10^7^ particles mL^−1^) with a bacterial isolate (final conc. 10^6^ cells mL^−1^) was incubated for 3 h at RT in the dark on a shaker (NR-80; Taitec, 100 rpm) (bacterial addition treatment). Also, PSBs (final conc. 10^7^ particles mL^−1^) in < 0.02-μm-filtered FASW without bacteria were incubated as a control (PSB-only control). Then, samples were filtered with 0.4 µm polycarbonate filters (25 mm diameter; Merck Millipore) with a vacuum of less than 150  mmHg. Subsequently, 1 mL of <0.02-μm-filtered FASW with formaldehyde (final conc. 2%) was filtered to wash and fix samples. After drying, the filter was stored at −20°C until further analysis. Filters were coated with Au and attached to a brass base with carbon tape and observed by SEM under 5 kV. Digital images were captured, and attached PSBs on bacterial cells were counted manually on the screen with 20 areas for each sample.

### Statistical analysis

We used SigmaPlot 14.5 (Systat Software, Inc.) for Kruskal-Wallis one-way analysis of variance on ranks, after which Dunn’s all pairwise multiple comparison test was used to compare values of surface properties among bacterial species. Differences of bacterial size and surface properties among NBA and isolates were tested with Welch’s *t*-test using Microsoft Excel (Excel 2021; Microsoft). We used SigmaPlot 14.5 for Spearman rank order correlation and power regression analyses between surface properties and nanoparticle scavenging.

The bootstrap method was performed using Google Colaboratory (Python in the browser). First, data sets for bacterial surface properties (Young’s modulus or adhesiveness) and scavenged nanoparticle abundance (VLPs or PSBs) were provided. Random sampling with replacement was performed to create 100 data sets. Then, for each data set, the constants (*a*) and slopes (*b*) were determined by performing a power regression (y = *a*x*^b^*).

## RESULTS AND DISCUSSION

We employed AFM to measure Young’s modulus and adhesiveness of bacterial surfaces in seawater. The advantage of AFM is its ability to operate under conditions that closely mimic marine environments. We found that fixing and drying bacteria significantly altered Young’s modulus and adhesiveness, increasing both by ca. 2–10 times after 2% formalin fixation and drying (data not shown), consistent with studies on mammalian cells (46, 47). Due to technical limitations, i.e., the difficulty of maintaining surface properties over a certain period for sample analysis, our data set consists solely of coastal natural bacterial assemblages and isolates and does not include pelagic or deep-sea bacteria.

Moreover, because (i) measurements of bacterial surface properties were obtained by pressing an AFM probe approximately tens of nanometers into the sample and retracting it, (ii) many marine bacteria secrete capsules and possess extracellular structures (48, 49), and (iii) the height of the capsule-like material protruding from the cell surface was measured, allowing us to predict that its thickness in our samples ranged from a few tens to several hundreds of nanometers (Fig. S1), we believe that our AFM results likely represent a combination of outer membranes themselves and external structures (such as polysaccharides, proteins, lipids, and capsules).

Additionally, since AFM measurements were performed within 3–9 h after sample preparation, cellular changes may have occurred during this period. Therefore, we note that data of bacterial surface property may include time-dependent shifts as well as intercellular differences.

While these experimental biases may affect the range and errors of the results, investigating the surface properties, a largely unexplored bacterial characteristic, and their relationship with nanoparticle scavenging is meaningful for deepening our understanding of bacterial roles in marine biogeochemical cycles. This, therefore, supports the broader implications of our study.

### Bacterial surface properties are highly variable in seawater

**Fig 1:**
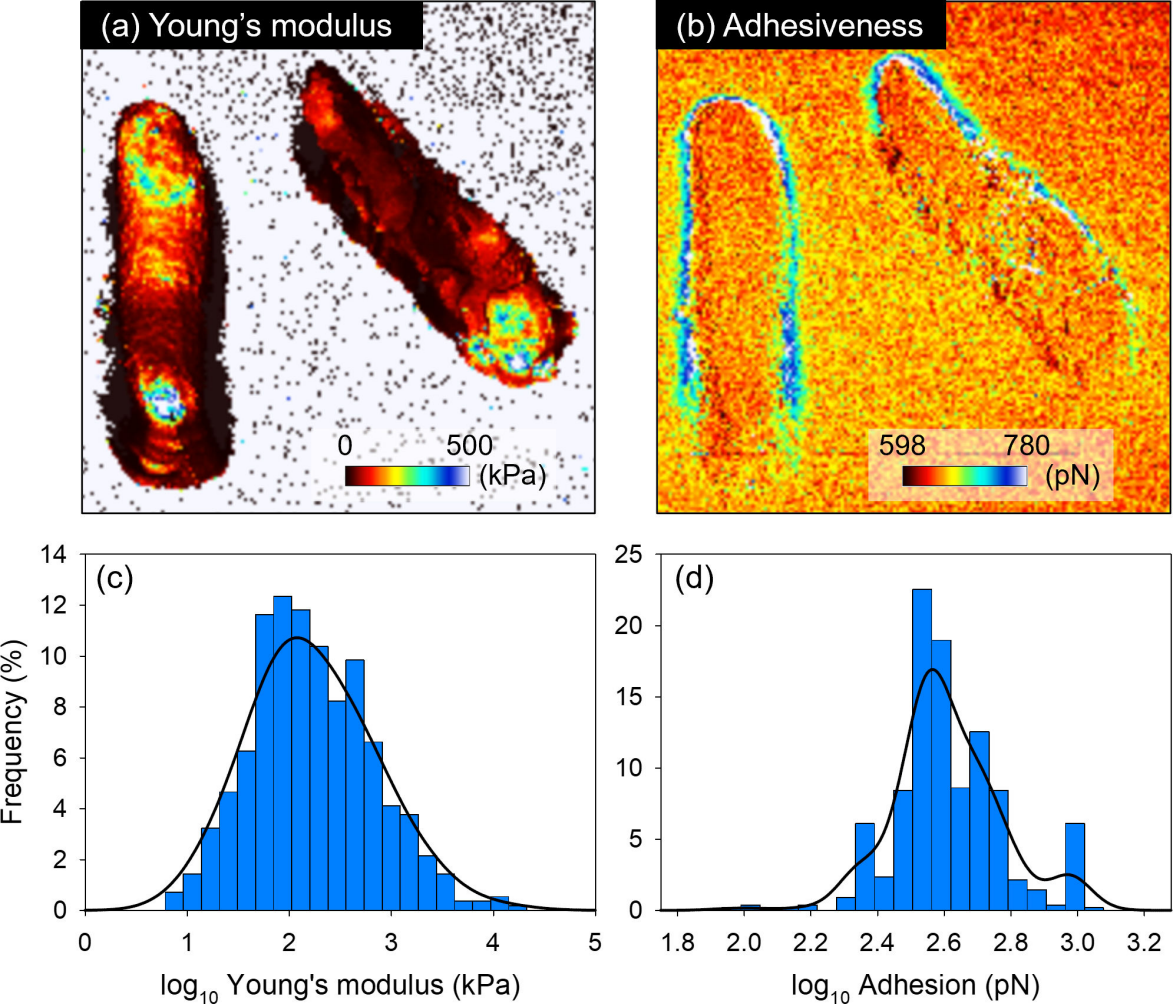
Representative AFM images of surface property measurements and distribution of surface properties of marine bacteria. (**a and b**) Surface property measurements of a marine bacterial isolate (*Vibrio coralliilyticus strain RE98*) (Images are 3.25 × 3.25 µm^2^; each pixel is 20 × 20 nm^2^). (**c and d**) Histograms and kernel density estimation of surface properties of bacteria isolated from coastal seawater, Okinawa, Japan. Values are expressed as individual cell means, and number of analyzed cells are 559.

Surface properties of natural bacterial assemblages (NBA) collected in coastal waters and marine bacterial isolates (total 559 cells) were determined using AFM (Fig. 1; Fig. S2). Surface characteristics exhibited unimodal distributions on a logarithmic scale. Young’s modulus values of individual cells ranged from 6 to 21,000 kPa (mean ± standard deviation [SD]: 500 ± 1,400 kPa), spanning four orders of magnitude. Young’s modulus for 85% of cells was between 25 and 1,000 kPa (Fig. 1a and c). Adhesiveness spanned two orders of magnitude, from 86 to 1,200 pN (440 ± 180 pN), with 85% of cells falling between 260 and 860 pN (Fig. 1b and d). Adhesiveness was relatively consistent among cells, whereas Young’s modulus exhibited relatively large variations, even on a single cell (Fig. 1a and b). This variability of Young’s modulus is likely attributable to components of cell walls such as lipopolysaccharides, peptidoglycans, and lipid bilayers (16) and the presence of nucleoids and heterogeneous distribution of biomolecular condensates in bacterial cells (37). In our data set, no clear relationship was observed between high Young’s modulus values and the spatial location of measurements on the cells (Fig. S3). Further validation is required, but it is possible that the thickness and structure of cell surface material having the same composition significantly affect Young’s modulus while having little impact on adhesiveness. We note that values observed here fall roughly within reported values for Young’s modulus (40–769,000 kPa) and adhesiveness (200–8,500 pN) of non-marine bacteria (Table S1).

Observed cell sizes ranged from 0.31 to 1.9 µm in ESD (mean ± SD, 0.93 ± 0.21 µm; number of analyzed cells = 559), and cell peak height and lengths of long and short axes ranged from 65 to 1,800 nm (780 ± 250 nm), 230 to 4,100 nm (1,600 ± 600 nm), and 180 to 2,100 nm (680 ± 220 nm), respectively (Table 1). The ESD and cell peak height were similar for NBA and isolates, but NBA cells were 0.7 times shorter on the long axis and 1.3 times longer on the short axis, demonstrating that NBA cells are relatively round, whereas isolates are relatively rod-shaped (Table 1, representative images shown in Fig. 1; Fig. S2, S4 through S7). Because NBA includes various bacterial species, a simple comparison between NBA and isolates requires caution. However, even though NBA and isolates had similar adhesiveness (1.05 times difference), Young’s modulus of NBA was 11 times higher. A relatively high negative correlation was found between the long: short axis ratio and Young’s modulus, suggesting that this ratio influences Young’s modulus, but not adhesiveness (Table S6). Although this result contradicts reports indicating that higher amounts of cytoskeletal proteins lead to longer and stiffer bacterial cells (50, 51), it suggests that other factors, including bacterial species, cell age, and non-bacterial substances, such as nonspecific attached mineral particles [Young’s modulus: 2–480 MPa (52)] in seawater and other chemical and environmental parameters, may also influence Young’s modulus. These factors should be explored further in future studies.

**TABLE 1:** Summary of measured bacterial size and surface properties in this study^*a*^

	ESD (μm)	Peak height (nm)	Long axis (nm)	Short axis (nm)	Long : short axis ratio	Long : height axis ratio	Young’s modulus (kPa)	Adhesiveness (pN)
ALL (number of analyzed cells = 559)	0.93 ± 0.21	780 ± 250	1,600 ± 600	680 ± 220	2.6 ± 1.2	2.4 ± 2.0	500 ± 1,400	440 ± 180
Natural bacterial assemblages (NBA) (number of analyzed cells = 57)	0.89 ± 0.27	760 ± 270	1,100 ± 310^*b*^	860 ± 330^*b*^	1.5 ± 0.80^*b*^	1.7 ± 0.92^*b*^	2,700 ± 3,500^*b*^	460 ± 170
Isolates (number of analyzed cells = 502)	0.93 ± 0.20	780 ± 240	1,700 ± 600	660 ± 190	2.7 ± 1.2	2.5 ± 2.0	250 ± 350	440 ± 180
Ratio (NBA : isolates)	0.95 ± 0.36	0.97 ± 0.46	0.67 ± 0.30	1.3 ± 0.63	0.56 ± 0.38	0.71 ± 0.69	11 ± 20	1.0 ± 0.57

^*a*^ Equivalent spherical diameter (ESD) was calculated from cell height images (see Materials and Methods). Values are mean ± standard deviation.

^*b*^ *P* <0.001; statistical differences among NBA and isolates were tested with Welch's t-test.

### Surface properties and nanoparticle scavenging by different bacterial species

**Fig 2:**
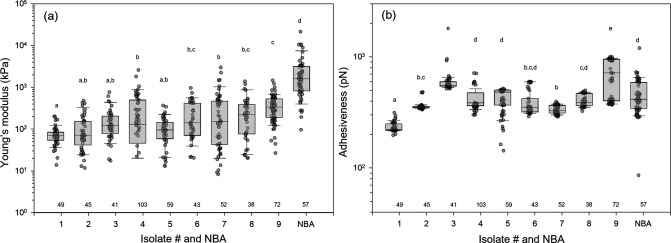
Surface property variation of bacterial isolates and natural bacterial assemblages (NBA). Box and whisker plots of surface properties, (**a**) Young’s modulus, (**b**) adhesiveness, for bacterial isolates. Boundaries of each box indicate the 25th and 75th percentiles. Error bars above and below boxes indicate the 10th and 90th percentiles, respectively. A line within a box marks the median. Statistical differences among isolates and natural assemblages were tested with one-way ANOVA on ranks and Dunn’s *post-hoc* test. Different letters indicate that mean values differed significantly (*P* < 0.05). Numbers of analyzed cells are shown for each isolate and NBA. Table S2 shows which isolate corresponds to each Isolate #.

We compared nanoscale surface properties of bacterial isolates with those of NBA to investigate the species-specific difference (Fig. 2). For Young’s modulus, species-specific mean values ranged from 75 to 2,687 kPa, with a 36-fold range of variation (Fig. 2a). Adhesiveness ranged from 230 to 690 pN with 3.0-fold variation (Fig. 2b). Bacterial species used in this study were randomly isolated from coastal communities. Although there are only nine isolates, it is evident that both Young’s modulus and adhesiveness exhibit a wide range, even at the genus level, as seen in *Vibrio* Isolates #1 and #9 (Fig. 2a and b; Table S2). This indicates that while each bacterial species has specific surface properties, these values can vary largely even among those sharing the same genes, highlighting the need for further analyses at the single-cell level.

**Fig 3:**
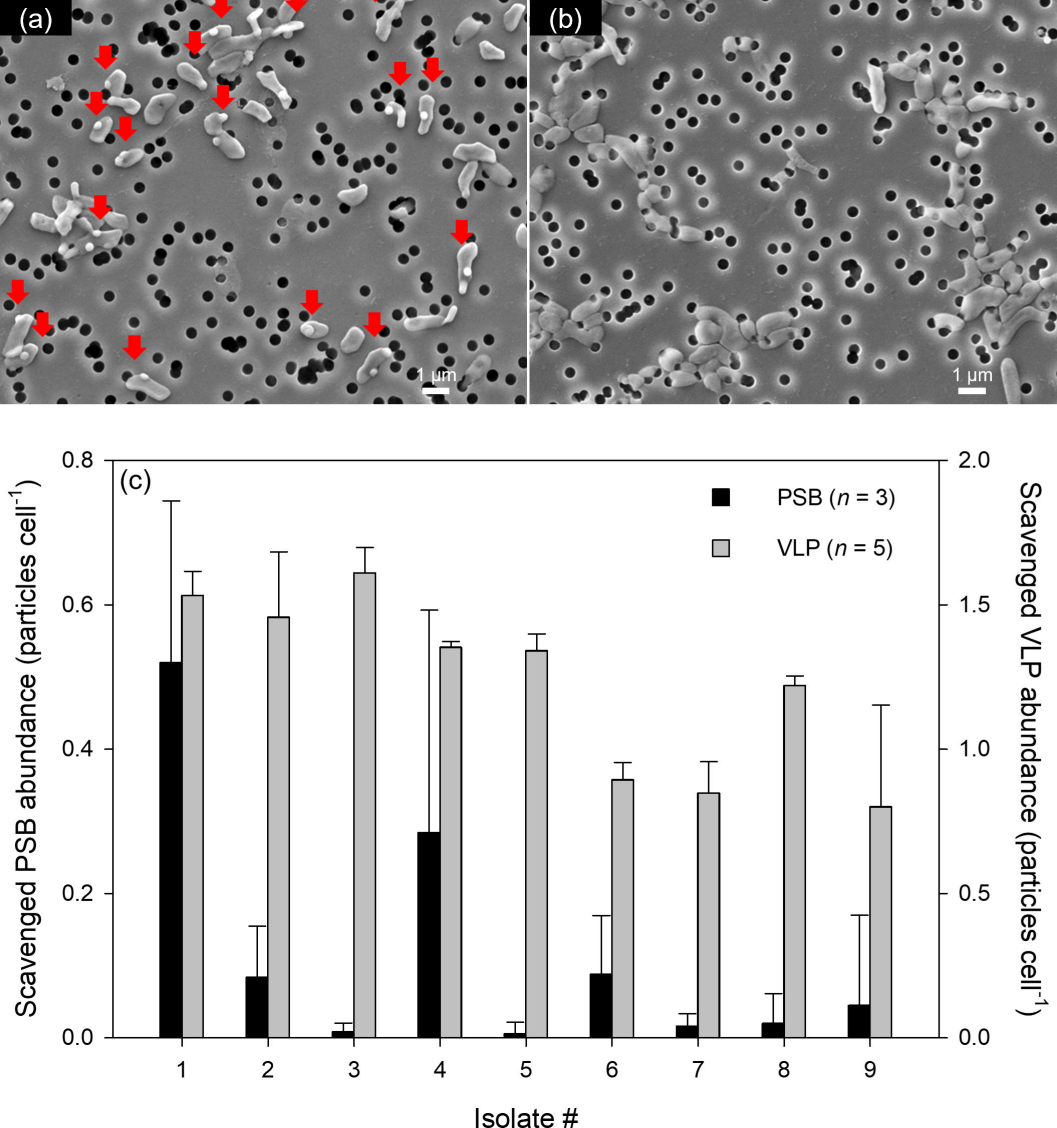
Nanoparticle scavenging of individual isolates. Representative images of bacterial cells, (**a**) isolate #4 and (**b**) isolate #8, with or without polystyrene beads (PSB) attachments (red arrows) observed with scanning electron microscopy. (**c**) Scavenged nanoparticle abundance for each isolate. PSB and virus-like particles (VLP) were used as model nanoparticles. Values are expressed as means ± standard deviations. Table S2 shows which isolate corresponds to each Isolate #.

We also quantified nanoparticle scavenging using PSBs and VLPs as model nanoparticles in coincubation experiments with each isolate (Fig. 3). Mean scavenging values for PSBs and VLPs ranged from 0.0052 to 0.52 particles cell⁻¹ and 0.80 to 1.6 particles cell⁻¹, with 100-fold and 2.0-fold variation among species (Fig. 3c). Although quantitative methods for scavenged VLPs and PSBs are different, which may introduce some bias, VLPs attach to bacteria ca. 1.5–300 times more efficiently than PSBs. This result may reflect differences in size, shape, and surface structure and composition between VLPs and PSBs (15, 53). Also, it is possible that natural polymers in the VLP suspension (see Materials and Methods) increase the adhesiveness of VLPs, promoting VLP attachment to bacteria (45, 54). We note that since cell size varies across bacterial isolates (with about a twofold difference in mean cell diameter, data not shown), comparing scavenged nanoparticle abundance “per unit area” instead of “per cell” may provide a more accurate comparison across isolates. However, even after recalculating and plotting accordingly (Fig. S8 and S9), the results do not show significant differences.

**Fig 4:**
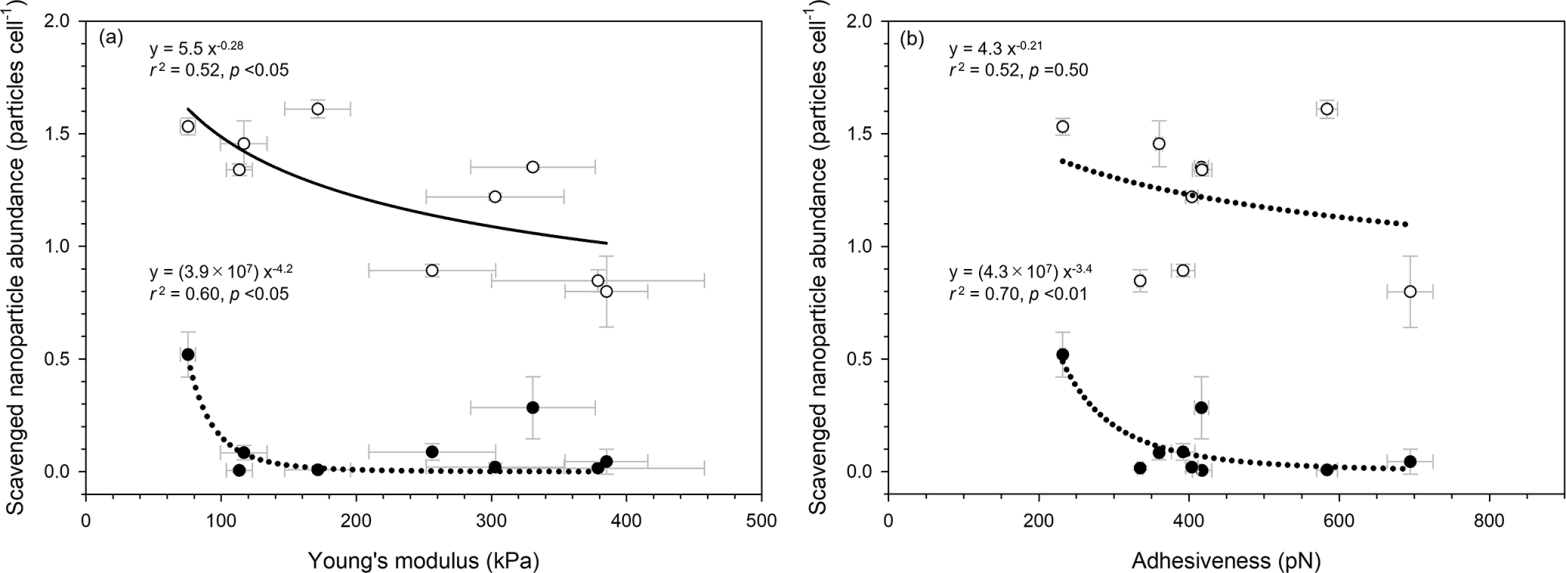
Relationship between bacterial surface properties, (**a**) Young’s modulus, (**b**) adhesiveness, and nanoparticle scavenging. This figure includes data from nine isolates for scavenging virus-like particles (VLPs; white plots) and polystyrene beads (PSBs; black plots). Each plot shows the mean ± standard error (*n* = 5 and *n* = 3 for VLP and PSB scavenging, respectively, and number of analyzed cells = 38–103 for surface properties). The power regression (solid line) and trend lines (dotted lines) (see discussion) are obtained by nonlinear curve fitting, and the coefficient of determination (*r^2^)* and statistical significance (*p*) are given for each relationship.

Using data from isolates (Fig. 2 and 3), we illustrated relationships between bacterial surface properties and nanoparticle scavenging (Fig. 4). This figure shows that for both PSBs and VLPs, Young’s modulus and adhesiveness are negatively related to nanoparticle scavenging (*P* < 0.05), except that the relationship between adhesiveness and scavenged viral abundance is negative, but insignificant (*P* = 0.50). As regression plots for PSBs, i.e., black plots in Fig. 4, appear to be influenced by a single data point at the leftmost end, we conducted a validation of our results using bootstrapping (55) (also see Materials and Methods and Fig. S10). This revealed that, while the relationship between Young’s modulus and VLP scavenging is consistently (100%) negative, other relationships showed that a negative correlation did not occur in 8%–21% of cases. Therefore, we conclude that in our data set, a negative correlation was observed only between Young’s modulus and VLP scavenging, but no other significant relationships were observed. A significant negative correlation was found between Young’s modulus and VLP scavenging, but not with PSB. This discrepancy may be attributed to several differences in the properties of VLPs and PSBs, such as size, shape, surface structure, and composition (15, 53). VLPs may have complex surface structures, including proteins, lipid membranes, and tails, enabling specific interactions with cellular receptors (44, 56). These features may influence their attachment to bacteria. In contrast, PSBs have smooth, synthetic surfaces lacking biological specificity, likely reducing their attachment potential. In addition, the lower number of scavenged PSBs compared to VLPs may have been insufficient to establish a meaningful correlation with Young’s modulus.

The tendency for lower Young’s modulus to facilitate VLP scavenging could be explained by the higher deformability of cells with lower Young’s moduli, resulting in increased attachment area and van der Waals forces. This is consistent with the observation of a previous study showing that bacteria with greater deformability are more likely to adhere to glass surfaces (12). However, as Young’s modulus values of NBA deviate from those of isolates (Fig. 2a), we note that further validation is necessary before applying the results to field samples.

There was no significant relationship between adhesiveness and nanoparticle scavenging in our data. Generally, factors influencing bacterial adhesiveness in liquid include charge, hydrophobicity, the presence and quantity of substances such as extracellular polymeric substances (EPS), and fine structure of bacterial surfaces (57–60). Additional experiments were then conducted to identify factors related to adhesiveness, such as charge, hydrophobicity, and EPS amounts on the bacterial surface. However, no significant correlation was observed (see the supplemental methods, Table S5, and Fig. S11 and S12). These results suggest that other substances, e.g., specific proteins (21), or the presence and number of fine structures suitable for nanoparticle attachment (57) may be involved, but further investigation is needed. To comprehend mechanisms influencing nanoparticle scavenging, additional studies are required to examine relationships between these factors and nanoparticle scavenging among diverse nanoparticles and bacterial species in various environmental conditions at the single-cell level.

### Implications for nanoparticle-bacterial interactions, bacterial survival strategies, and carbon cycles

Previous studies have linked microbial physiological processes in the oceans to large-scale ecological dynamics by examining single-cell properties of marine microbes (61, 62). Our AFM data on nanoscale surface properties of marine bacterial surfaces suggest that in addition to roughness, Young’s modulus provides valuable insights into nanoparticle-bacteria interactions. While the significance of these surface characteristics in attachments between objects and elastic bodies has been well documented (10, 11), our study potentially extends this understanding to attachments between marine bacteria and nanoparticles. Given the fact that Young’s modulus varies greatly among non-marine archaea and gram-positive bacteria (22, 63), Young’s modulus may also influence nanoparticle attachment in other prokaryotes in seawater, similar to the gram-negative marine bacterial isolates we measured.

The finding that Young’s modulus of marine bacteria is related to their VLP scavenging as model organic nanoparticles suggests a trade-off, whereby bacteria optimize organic nanoparticle capture while minimizing viral infection, thereby enhancing bacterial survival. This trade-off aligns with the strategy hypothesis of SAR11, which exhibits lower surface hydrophobicity compared to other bacteria, avoiding predators while reducing organic matter attachment (64). Also, according to a previous report stating that 5%–70% of natural marine bacteria are motile (65), bacterial optimization of surface properties for organic nanoparticle capture may be related to bacterial motility. This strategy involves a trade-off between using energy for movement to increase the frequency of collisions with nanoparticles or conserving energy by remaining stationary. As bacteria are fundamental to biogeochemical cycling by degrading organic matter and facilitating nutrient regeneration (6), these trade-offs have significant implications for microbial food webs and ecosystem stability in marine environments. Further investigation into temporal changes in bacterial surface properties and their responses to viruses and other nanoparticles is necessary to understand their impact on bacterial survival strategies and the surrounding ecosystem.

A ~ fivefold increase in Youngʼs modulus results in an approximately 50% decrease in nanoparticle attachment (Fig. 4). While this change may seem modest, it could have significant ecological implications. Given the enormous number of bacteria in the ocean, even a ~ 50% reduction in nanoparticle attachment per single cell could translate into substantial impacts on marine biogeochemical cycles. Moreover, the variation in Young’s modulus of natural marine bacteria is much larger (3 orders of magnitude, Fig. 2a) than the ~fivefold range observed in our experimental isolates (Fig. 4), suggesting a potentially large effect of Young’s modulus on nanoparticle attachment in natural environments. Furthermore, since viruses are key drivers of bacterial mortality in marine environments (66), even a 50% variation in adsorption rate could substantially impact the microbial loop and associated biogeochemical cycles (67). While other nanoparticle removal pathways such as aggregation or abiotic degradation remain less understood (68), our findings highlight the significance of cell mechanical properties in shaping these processes.

In the surface layer of the ocean, concentrations of nanoparticles and bacteria are estimated to be 10^9^ nanoparticles mL^−1^ (5) and 10^7^ cells mL^−1^ (69), respectively. Based on our study (scavenging rate: 10^0^–10^1^ nanoparticles cell^−1^ d^−1^; calculated from VLP scavenging in 3 h), we estimate that approximately 1%–10% of nanoparticles (10^7^–10^8^ nanoparticles mL^−1^) can be removed from seawater every 24 h. Since dissolved organic carbon (DOC) accounts for the majority (>90%) of carbon in the ocean, and 10%–40% of DOC has been reported as nanoparticles (colloidal particles) (70), it is estimated that 0.1%–4% of DOC per day is utilized by bacteria via nanoparticles. This process likely transports organic carbon to higher trophic levels through predation of bacteria by protists and zooplankton (6) and contributes to remineralization of organic carbon, degrading it into inorganic forms and making it available for primary producers (71). This may also influence the formation of larger aggregates and vertical carbon export by regulating nanogel assembly (72, 73). Thus, bacterial strategies for modulating surface properties and their effects on nanoparticle capture efficiency, as highlighted in this work, have significant implications for large-scale ocean biogeochemical processes (1, 74, 75).

## Data Availability

Data for bacterial and nanoparticle variables used in this study are available in Table 1 and Table S3 through S5. Other data supporting findings of this study are available from Y.Y. upon request.

## SUPPLEMENTAL MATERIAL

Supplemental material aem.01049-25-s0001.pdf Supplemental methods, Tables S1 to S6, and Figures S1 to S12.

ASM does not own the copyrights to Supplemental Material that may be linked to, or accessed through, an article. The authors have granted ASM a non-exclusive, world-wide license to publish the Supplemental Material files. Please contact the corresponding author directly for reuse.
