# Supplementary material for: Organic particle scavenging by marine bacteria: influences of bacterial nanoscale surface properties
Source: Appl Environ Microbiol. 2025 Jun 16;91(7):e01049-25. doi: 10.1128/aem.01049-25 (PMC12285227; doi:10.1128/aem.01049-25)
Supplement: Supplemental material — Supplemental methods, Tables S1 to S6, and Figures S1 to S12. [file aem.01049-25-s0001.pdf]

Supplemental Material of

Organic particle scavenging by marine bacteria: Influences of bacterial nanoscale surface properties

Yosuke Yamada[1,2,3*], Toshiaki Mochizuki[3], Nirav Patel[4], Farooq Azam[4], Hideki Fukuda[5], Toshi Nagata[5], Satoshi Mitarai[3,6]

[1] Kochi Institute for Core Sample Research, Institute for Extra-cutting-edge Science and Technology Avant-garde Research, Japan Agency for Marine-Earth Science and Technology (JAMSTEC), Nankoku, Kochi, Japan
[2] Advanced Institute for Marine Ecosystem Change (WPI-AIMEC), JAMSTEC, Yokohama, Kanagawa, Japan
[3] Okinawa Institute of Science and Technology Graduate University, Onna, Okinawa, Japan
[4] Scripps Institution of Oceanography, La Jolla, CA, USA
[5] Atmosphere and Ocean Research Institute, The University of Tokyo, Kashiwa, Chiba, Japan
[6] Super-cutting-edge Grand and Advanced Research (SUGAR) Program, Institute for Extra-cutting-edge Science and Technology Avant-garde Research (X-star), Japan Agency for Marine-Earth Science and Technology (JAMSTEC), Yokosuka, Kanagawa, Japan

*** Correspondence:** Yosuke Yamada
yamadayo@jamstec.go.jp

This file includes:

Materials and Methods
Tables S1–S6
Figs. S1–S12
References

Materials and Methods

Chemical properties of bacterial surface

Hydrophobicity

Cell surface hydrophobicity was determined by a bacterial adherence-to-hydrocarbons assay (Rosenberg et al. 1980; Lemke et al. 1995). Briefly, 70 µL of n-hexadecane was added to 630 µL of washed and concentrated bacterial isolate (see Methods) and vortexed for 2 min. Controls received 0.02 µm filtered, autoclaved artificial seawater (<0.02 µm-filtered FASW) instead of bacterial isolate. After 15 min at room temperature in the dark, 300 µL of the aqueous phase was transferred into a 96-well plate. The optical density at 600 nm (OD600) was measured by spectrophotometry (Varioskan Flash; Thermo Fisher Scientific, Massachusetts, USA) and hydrophobicity (%) was calculated using the following equation:

Hydrophobicity (%) = 100 × [1 – (OD600 in an aqueous phase after treatment / OD600 in an original bacterial suspension)].

Extracellular polymeric substances (EPS)

EPS (transparent exopolymer particles [TEP] and Coomassie Brilliant Blue stained particles [CSP]) on bacterial surfaces were removed by following previous papers (Vandevivere and Kirchman 1993; Liu et al., 2007). Briefly, washed, concentrated bacterial isolates (see Methods) were sonicated for 20 s at the setting ca. 1.7/10 (50W, 20kHz, ultra sonic homogenizer UH-50 with MS-3 attachment; SMT, Tokyo, Japan). Most cell capsular material, e.g., TEP and CSP, can be removed from the bacteria surface using this method without causing cell lysis [<10% of total cells; Vandevivere and Kirchman (1993)]. Bacterial cells were removed by centrifugation (4000×g, 8 min, 25°C). Then the supernatant (containing TEP and CSP) was filtered through 0.2-µm polycarbonate filters with a vacuum of <150 mmHg. Filtered (0.02 µm), autoclaved artificial seawater was used instead of bacterial suspension as a control. These filters stained with either 0.02% Alcian blue (8GX; Sigma-Aldrich) in 0.06% acetic acid for TEP or 0.04% Coomassie Brilliant Blue (G-250; SERVA electrophoresis) dissolved in <0.02 µm-filtered FASW for CSP. Excess dye was washed with Milli-Q water. Filters were frozen at -20°C prior to colorimetric determination in an onshore laboratory. For TEP measurements, filters were soaked in 6 mL of 80% sulfuric acid for 2 h at 25 °C on a shaker (BR-33FL; Taitec, 200

rpm), and absorbance at 787 nm was measured using a spectrophotometer (Passow and Alldredge 1995).

For CSP measurements, filters were soaked in 4 mL of 50% isopropyl alcohol with 3% SDS. The vials were shaken at 200 rpm (BR-33FL; Taitec, 200 rpm), at 37°C, in the dark for 2 hours and absorbance was measured at 615 nm using a spectrophotometer (Cisternas-Novoa et al. 2014). TEP and CSP concentrations were calculated using a calibration factor determined with xanthan gum (for TEP) or bovine serum albumin (for CSP). TEP and CSP concentrations were expressed in terms of pg xanthan gum equivalent per cell (pg XGeq. $cell^{-1}$) and pg BSA equivalent per cell (pg BSAeq. $cell^{-1}$), respectively.

The effect of surface charge on nanoparticle scavenging

To investigate the effect of surface charge on nanoparticle scavenging, we used three bacterial isolates and surface-modified ($-COO^{-}$, $-NH_4^{+}$) polystyrene beads (PSBs) with a diameter of 100–300 nm (SPHERO carboxyl and amino fluorescent particles; Spherotech Inc.). For comparison, we also used non-surface-modified polystyrene beads with a diameter of 90–300 nm (SPHERO Fluorescent Particles; Spherotech Inc.). As described in the main text (see Materials and Methods), after sonicating and diluting the PSB, each PSB was incubated with (or without as a control) each bacterial isolate for 3 hours at room temperature in the dark on a shaker. The final concentrations of PSBs and bacteria were 10^7 particles mL^{-1} and 10^6 cells mL^{-1}, respectively. After incubation, samples were filtered using 0.4-μm polycarbonate filters (25-mm diameter; Merck Millipore) and fixed with 1 mL of <0.02-μm-filtered artificial seawater containing formaldehyde (final concentration: 2%). After drying, the filters were observed by scanning electron microscopy to count the PSBs attached to the bacterial cell surfaces.

Tables

Table S1. Values measured by atomic force microscopy (AFM) for Young's modulus and adhesiveness of non-marine bacteria in references*

Parameters	Order	Range or average	References
Young's modulus (kPa)	Alteromonadales	40–98,000	Gaboriaud et al., 2008
	Myxococcales	250	Pelling et al., 2005
	Enterobacteriales	500–221,000	Eaton et al., 2008; Cerf et al., 2009; Chen et al., 2009
	Bacillales	200–769,000	Francius et al., 2008; Kumar et al., 2009
Adhesiveness (pN)	Rhodospirillales	200	van der Aa and Dufrêne, 2002
	Myxococcales	2,500	Pelling et al., 2005
	Enterobacteriales	800–6,700	Thio and Meredith, 2008; Zhang et al., 2011
	Bacillales	1,440	Harimawan et al., 2011
	Burkholderiales	7,880	Harimawan et al., 2011
	Pseudomonadales	8,530	Harimawan et al., 2011

*Note that the culturing and measurement conditions, as well as the types of AFM probes used, vary across the literature, making simple comparisons difficult

Table S2. Bacterial isolate # and corresponding species or genus

Isolate #	Name of species or genus
1	*Vibrio sp.*
2	*Grimontia marina*
3	*Marinomonas communis*
4	*Vibrio coralliilyticus strain RE98*
5	*Alteromonas sp.*
6	*Marinomonas posidonica*
7	*Vibrio aestivus*
8	*Vibrio coralliilyticus strain OCN014*
9	*Vibrio coralliilyticus strain 58*

Table S3. Summary of bacterial surface properties variables for each isolate in this study

Bacterial isolate # and natural bacterial assemblages (NBA)	Young's modulus (kPa)			Adhesiveness (pN)			*n*
	Mean	Standard deviation	Standard error	Mean	Standard deviation	Standard error	
1	75	39	6	232	24	3	49
2	117	116	17	360	33	5	45
3	171	156	24	584	89	14	41
4	331	468	46	416	95	9	103
5	113	75	10	417	100	13	59
6	256	307	47	392	103	16	43
7	379	568	79	335	28	4	52
8	303	314	51	404	49	8	38
9	385	260	31	695	260	31	72
NBA	2687	3528	467	456	165	22	57

Supporting Information Table S2 shows which isolate corresponds to each Isolate #

Table S4. Summary of scavenged nanoparticle variables for each isolate in this study

Isolate #	Scavenged PSB abundance (particles $cell^{-1}$)				Scavenged VLP abundance (particles $cell^{-1}$)			
	Mean	Standard deviation	Standard error	*n*	Mean	Standard deviation	Standard error	*n*
1	0.520	0.224	0.129	3	1.532	0.084	0.037	5
2	0.084	0.071	0.041	3	1.456	0.228	0.102	5
3	0.008	0.013	0.007	3	1.610	0.089	0.040	5
4	0.284	0.309	0.178	3	1.352	0.021	0.010	5
5	0.005	0.016	0.009	3	1.340	0.059	0.026	5
6	0.087	0.082	0.047	3	0.893	0.061	0.027	5
7	0.016	0.018	0.010	3	0.847	0.109	0.049	5
8	0.020	0.042	0.024	3	1.220	0.034	0.015	5
9	0.044	0.125	0.072	3	0.799	0.354	0.158	5

Supporting Information Table S2 shows which isolate corresponds to each Isolate #

PSB: polystyrene beads; VLP: virus-like particles

Table S5. The hydrophobicity and the amount of extracellular polymeric substances of bacterial surfaces for each isolate

Isolate #	Hydrophobicity (%)				TEP (pg XGeq. cell^{-1})				CSP (pg BSAeq. cell^{-1})			
	Mean	Standard deviation	Standard error	*n*	Mean	Standard deviation	Standard error	*n*	Mean	Standard deviation	Standard error	*n*
1	21.3	4.5	1.5	9	5.7	0.3	0.2	3	1.2	0.1	0.0	3
2	17.1	8.9	3.0	9	5.5	0.7	0.4	3	1.3	0.2	0.1	3
3	21.8	14.3	4.8	9	3.2	0.3	0.2	3	0.8	0.1	0.0	3
4	17.6	9.3	3.1	9	4.0	0.4	0.2	3	0.9	0.1	0.1	3
5	19.9	17.5	5.8	9	4.9	1.8	1.0	3	1.3	0.5	0.3	3
6	20.8	9.1	3.0	9	2.4	0.7	0.4	3	0.6	0.2	0.1	3
7	9.8	10.6	3.5	9	10.2	1.1	0.6	3	2.7	0.3	0.1	3
8	14.9	9.3	3.1	9	8.7	1.8	1.1	3	2.4	0.5	0.3	3
9	22.2	4.5	1.5	9	9.9	1.4	0.8	3	2.2	0.3	0.2	3

Supporting Information Table S2 shows which isolate corresponds to each Isolate #

TEP: transparent exopolymer particles; CSP: Coomassie Brilliant Blue stained particles

Table S6. Spearman rank order correlation values between bacterial surface properties and cellular morphologies.

	ESD (μm)	Peak height (nm)	Long axis (nm)	Short axis (nm)	Long : short axis ratio	Long : height axis ratio
Young's modulus (kPa)	0.0	**-0.2**	**-0.2**	**0.3**	**-0.4**	0.0
Adhesiveness (pN)	**0.2**	0.0	**0.1**	**0.3**	**-0.1**	**0.2**

Number of analyzed cells was 559. Bold values were correlated significantly (p <0.05). ESD: equivalent spherical diameter

Figures

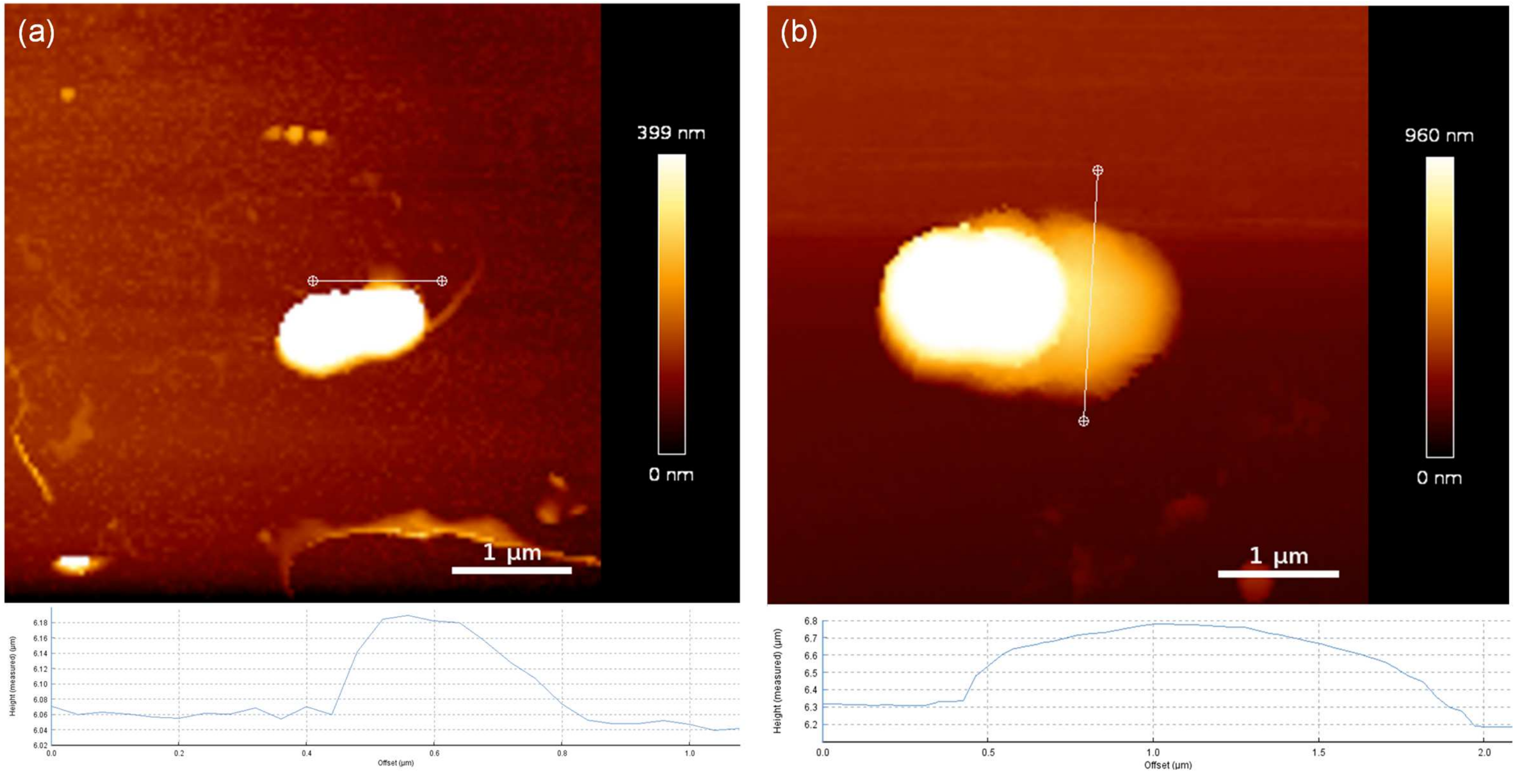

Fig. S1. Example of height measurement of capsule-like material protruding from bacterial cells by atomic force microscopy. (a) Isolate and (b) natural bacterial assemblages. In each image, the height profile along the white line in the upper image is shown in the lower image, with the thickness of the capsule-like material measured at approximately 120 nm and 500 nm, respectively.

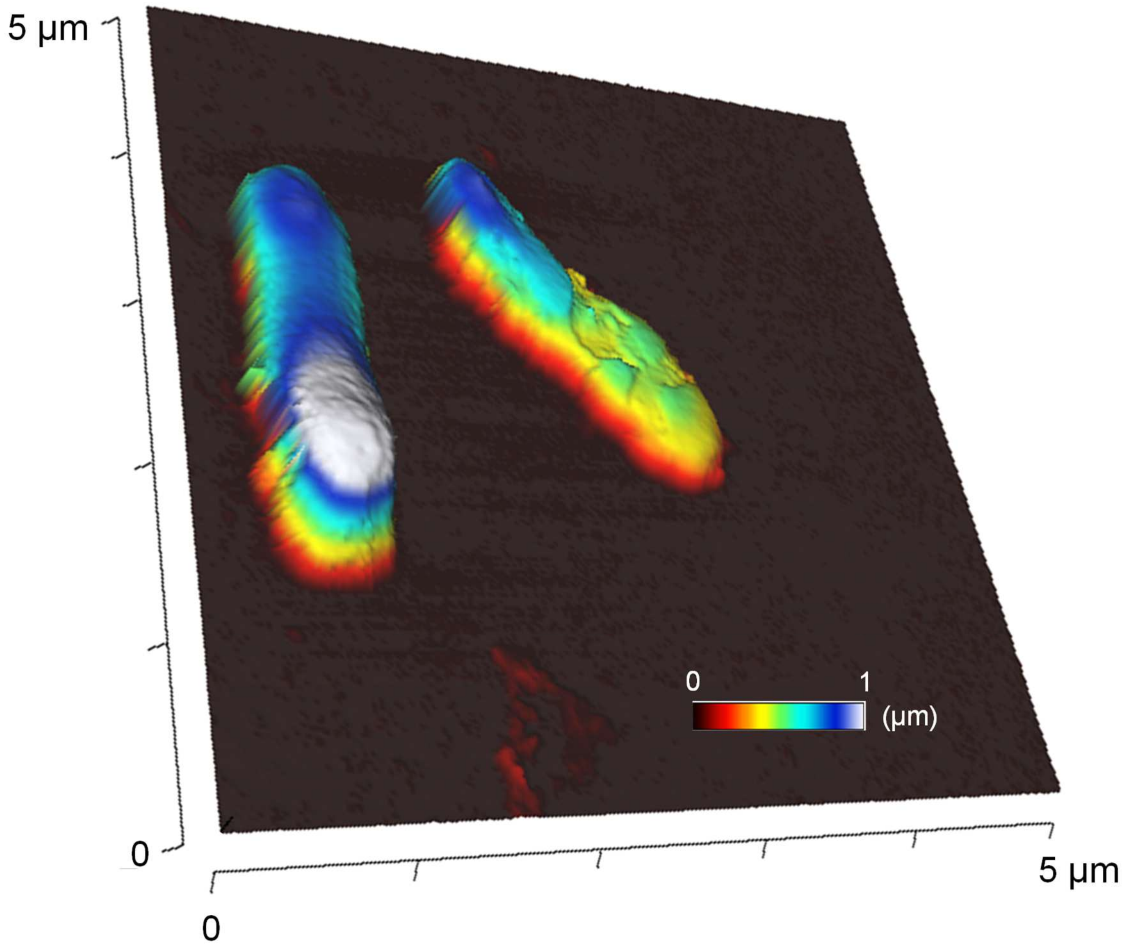

Fig. S2. An 3D height image of marine bacterial isolate (*Vibrio coralliilyticus strain RE98*) (Images are 5 x 5 µm^2; each pixel is 20 x 20 nm^2). Color contour shows the height of cells.

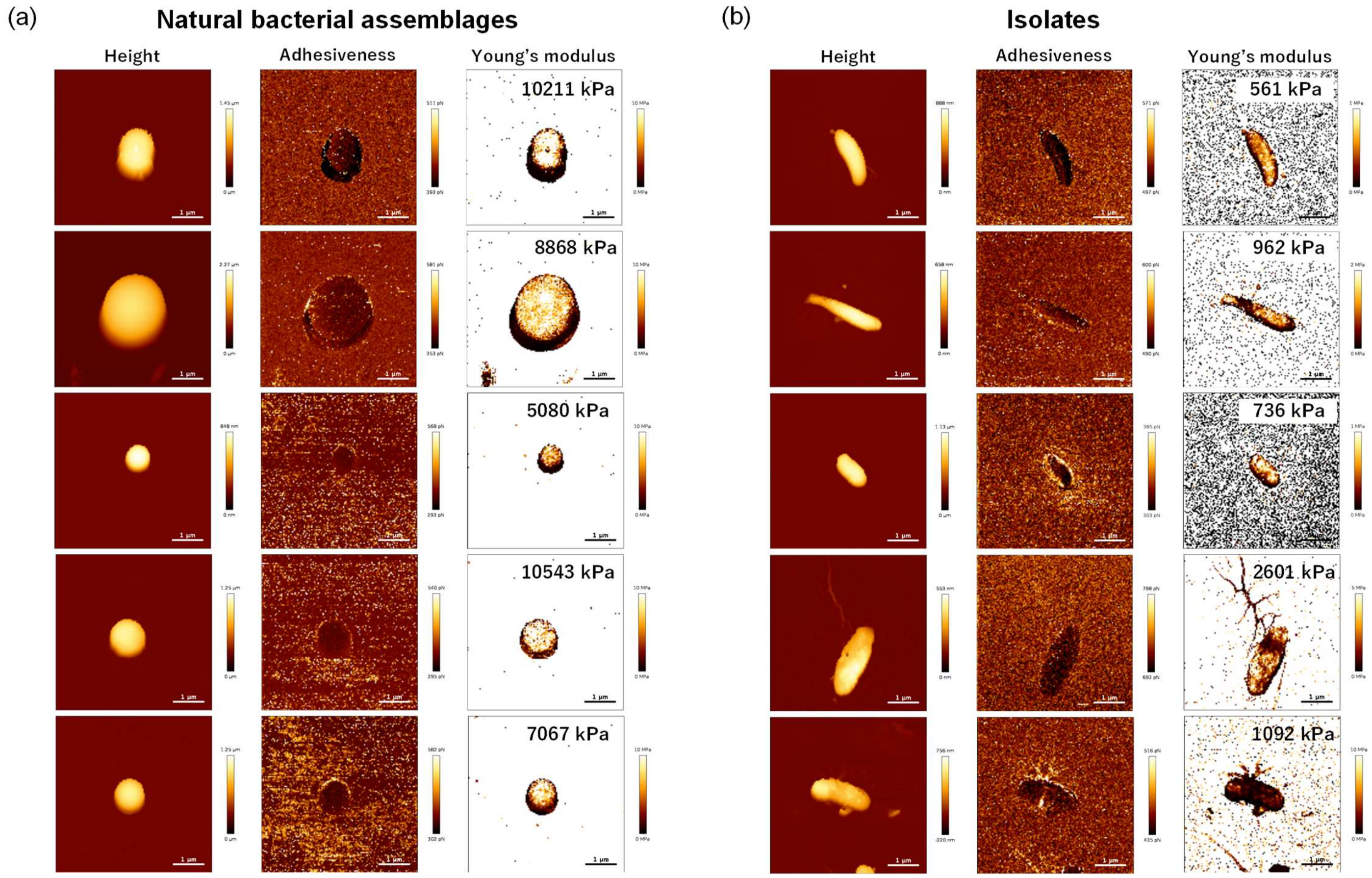

Fig. S3. Example images of bacterial cell morphology with extremely high Young's modulus, observed by atomic force microscopy. (a) Natural bacterial assemblages and (b) isolates. The mean Young's modulus values of each cell (measured in 1–5 areas, see Materials and Methods) are indicated in each figure.

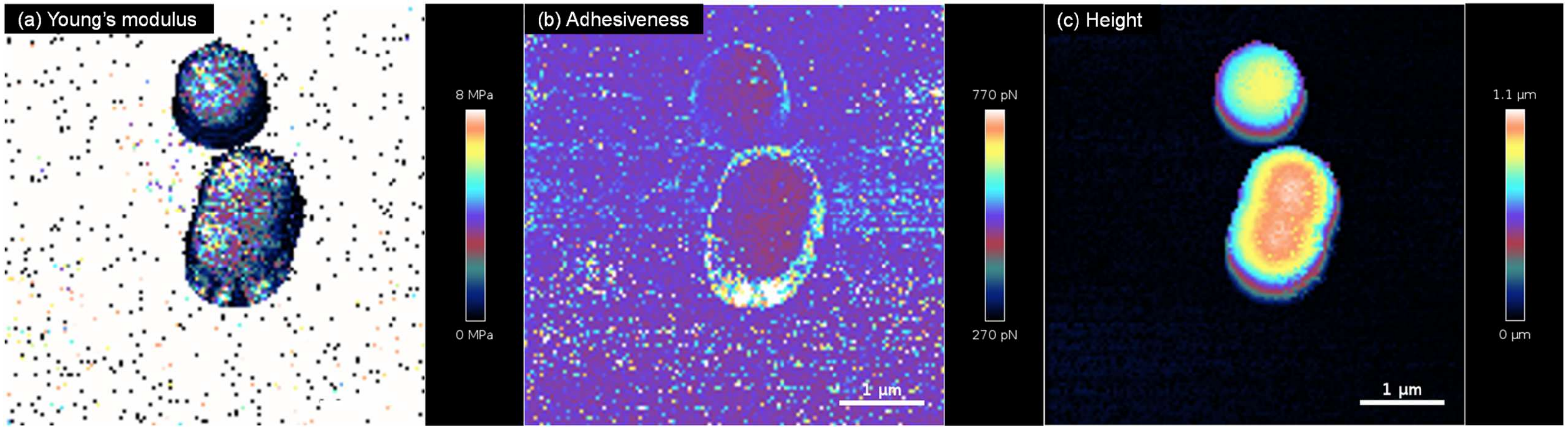

Fig. S4. Representative images of natural bacterial assemblages (NBA) by atomic force microscopy. (a-c) Surface property measurements of bacteria collected from coastal seawater, Okinawa, Japan (Images are 5 x 5 μm^2; each pixel is 40 x 40 nm^2). NBA cells had relatively smaller long: short axis ratios and more spherical shapes compared to rod-shaped isolates (see Fig. 1).

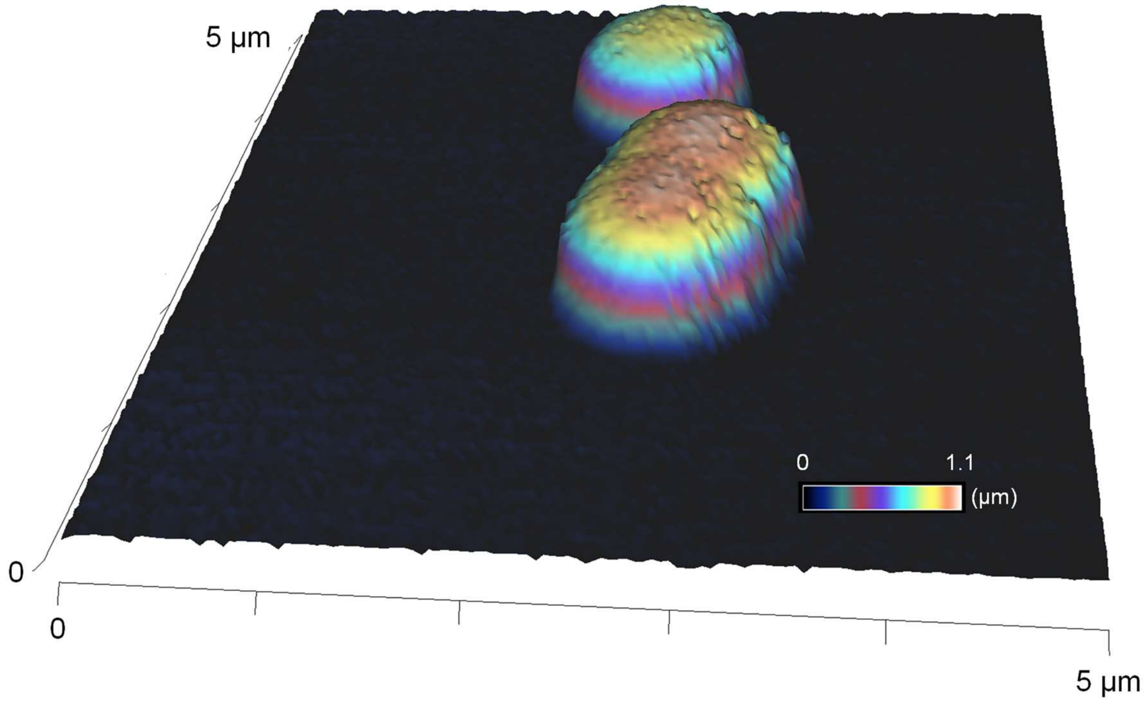

Fig. S5. An 3D height image of bacteria collected from coastal seawater, Okinawa, Japan (Images are 5 x 5 µm^2; each pixel is 40 x 40 nm^2). Color contour shows the height of cells.

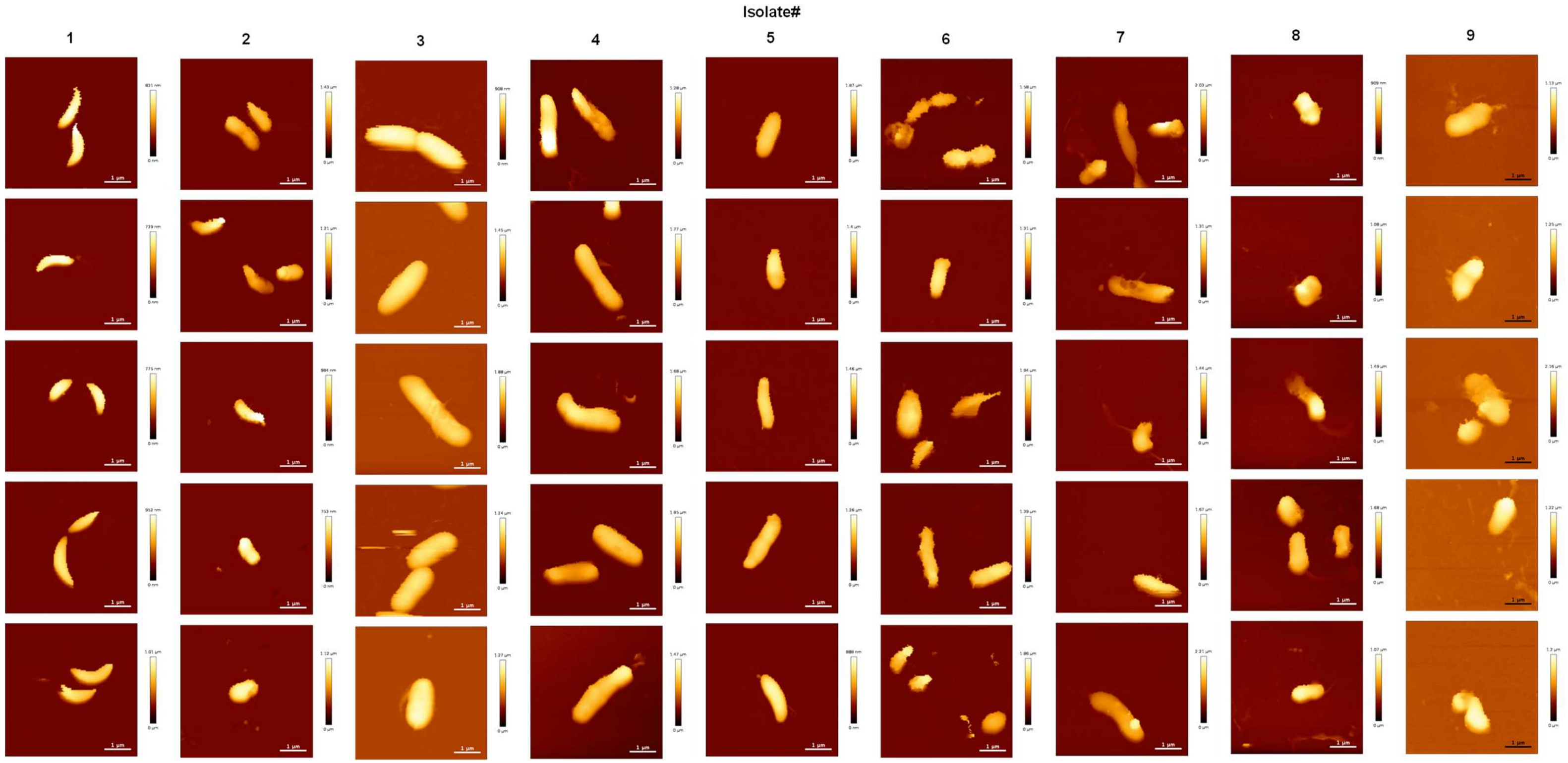

Fig. S6. Randomly selected atomic force microscopic images of bacterial isolates. The images show the height contour of bacterial cells. Each bacterial isolate is arranged in a single row and Supporting Information Table S2 shows which isolate corresponds to each Isolate #.

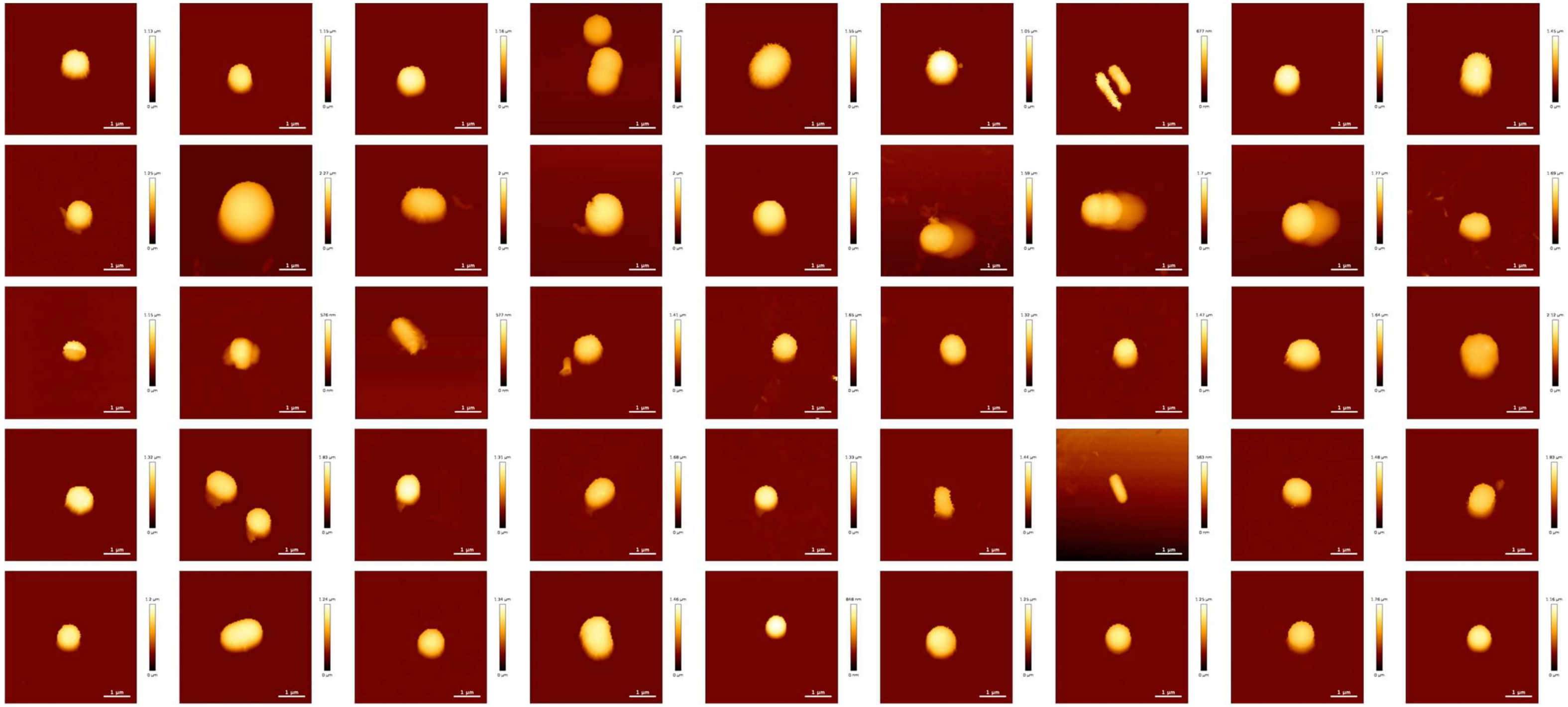

Fig. S7. Randomly selected atomic force microscopic images of natural bacterial assemblages (NBA). The images show the height contour of bacterial cells.

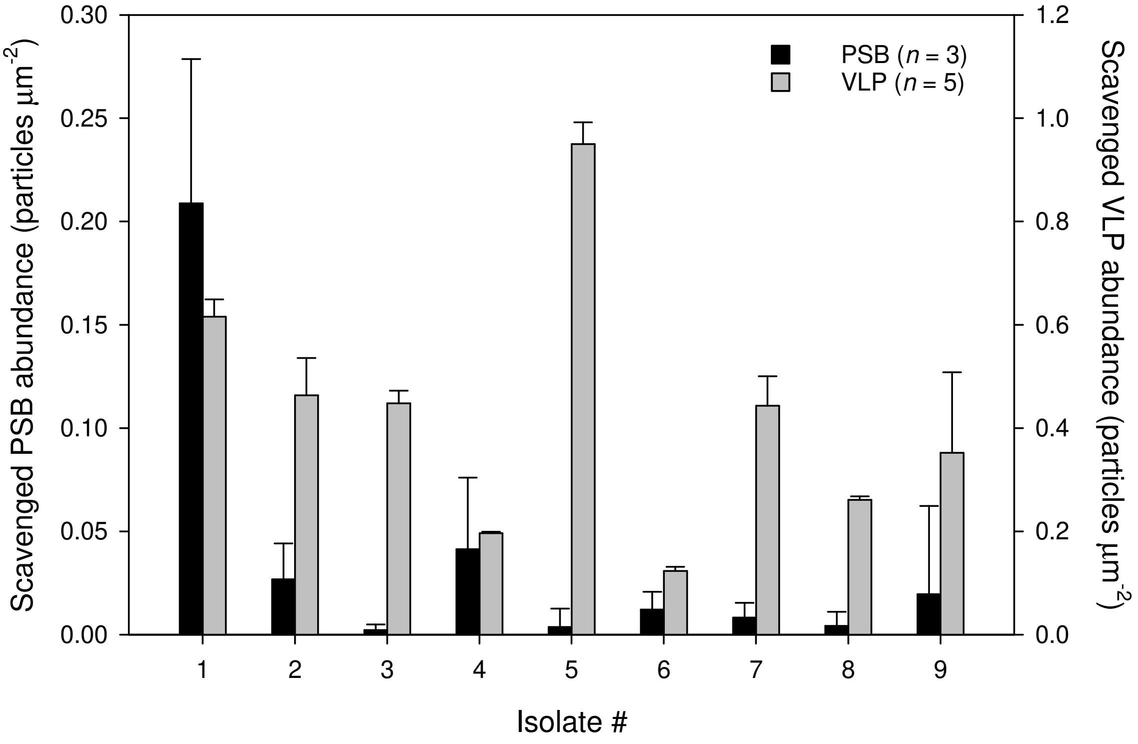

Fig. S8. The scavenged nanoparticle abundance for each isolate, normalized by the bacterial cell size. Values are means ± standard deviations. Supporting Information Table S2 shows which isolate corresponds to each Isolate #. PSB: polystyrene beads, VLP virus-like particles

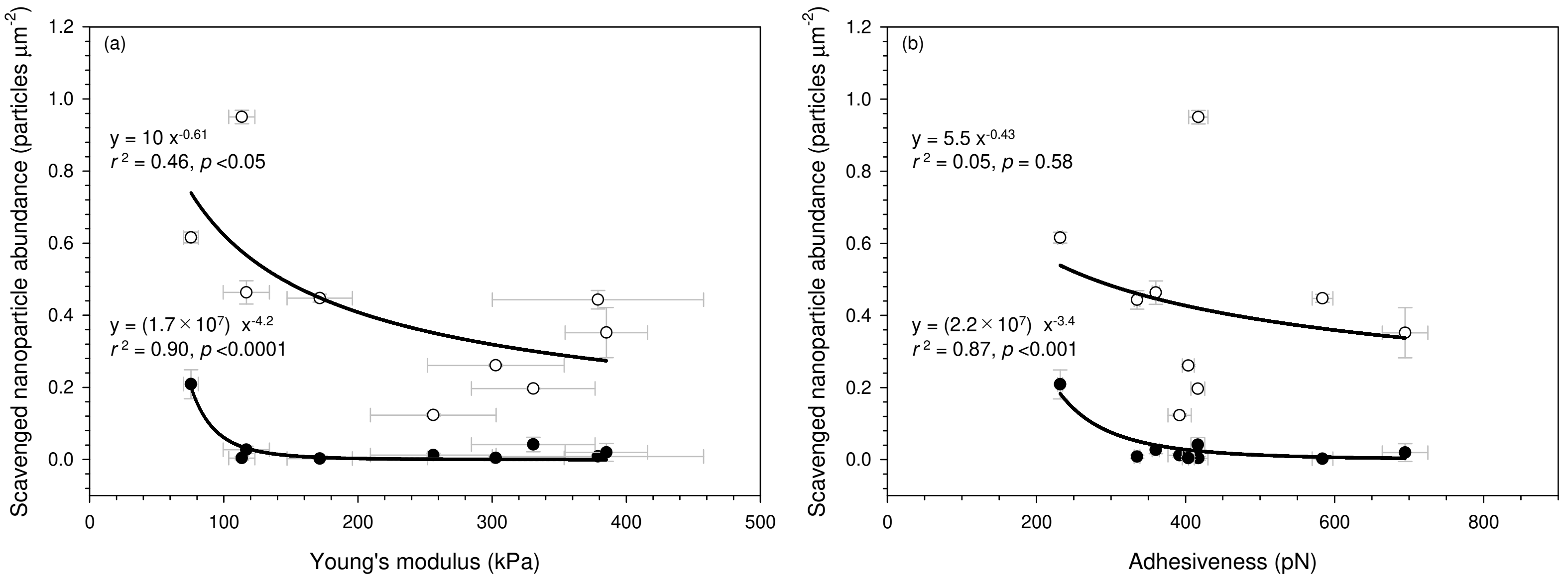

Fig. S9. Relationship between bacterial surface properties, (a) Young's modulus, (b) adhesiveness, and nanoparticle scavenging, normalized by the bacterial cell size. This figure includes data from nine isolates for scavenging virus-like particles (VLPs; white plots) and polystyrene beads (PSBs; black plots). Each plot shows the mean ± standard error (n = 5 and n = 3 for VLP and PSB scavenging, respectively, and number of analyzed cells = 38–103 for surface properties). Power regressions are obtained by nonlinear curve fitting, and coefficient of determination (r^2) and statistical significance (p) are given for each relationship.

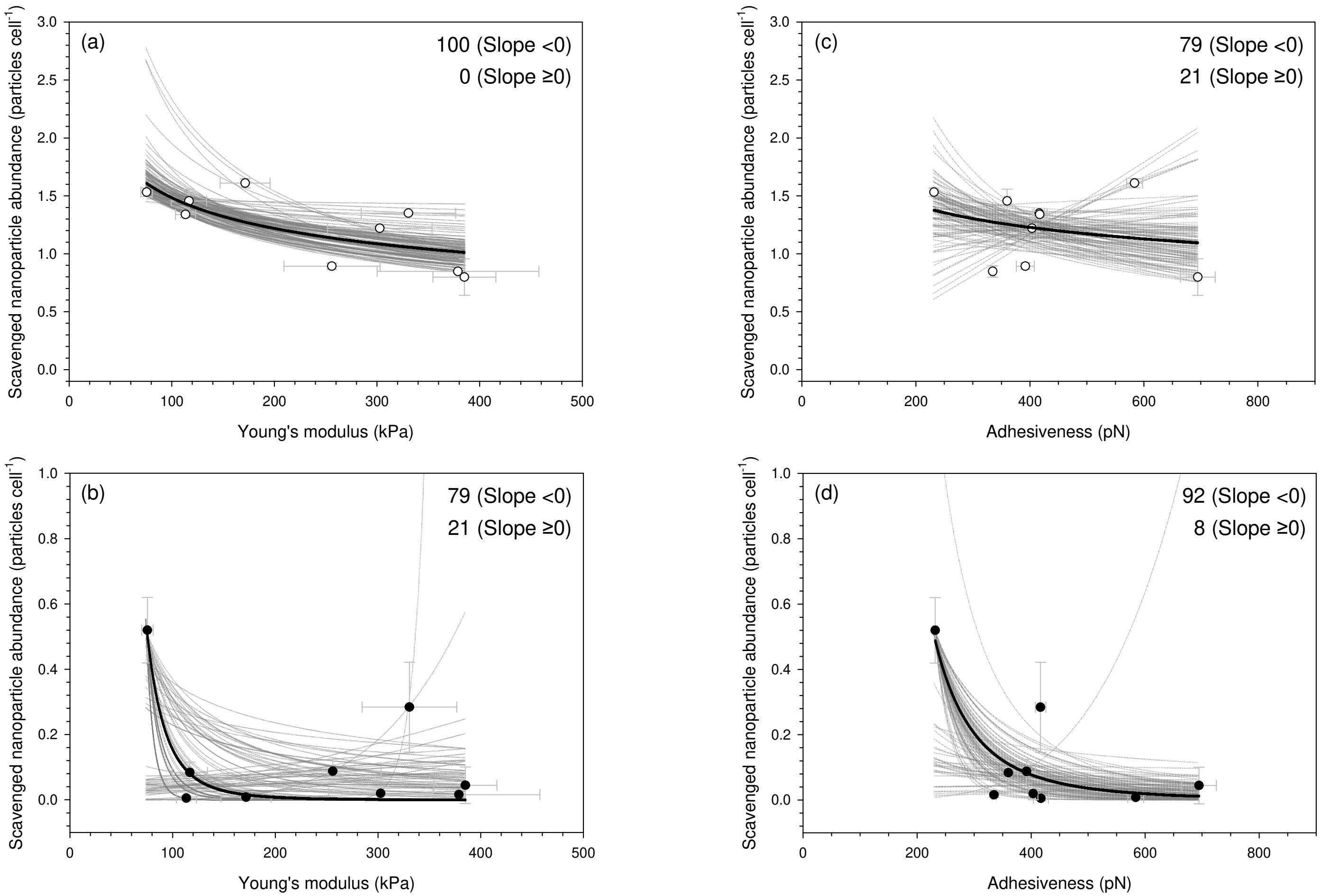
(a)
100 (Slope <0)
0 (Slope ≥0)
(b)
79 (Slope <0)
21 (Slope ≥0)
(c)
79 (Slope <0)
21 (Slope ≥0)
(d)
92 (Slope <0)
8 (Slope ≥0)
Scavenged nanoparticle abundance (particles cell^{-1})
Young's modulus (kPa)
Adhesiveness (pN)

Fig. S10. Relationship between bacterial surface properties and nanoparticle scavenging, along with regressions derived from 100-time bootstrap resampling. The regression lines from the bootstrap resampled datasets are shown in light gray. The relationship between Young's modulus and scavenged (a) virus-like particles (VLPs; white plots) / (b) polystyrene beads (PSBs; black plots), as well as the relationship between adhesiveness and scavenged (c) VLPs (white plots) / (d) PSBs (black plots), is shown. This figure includes the same plots and regressions as in Fig. 4. The numbers in the upper right indicate the frequency of negative and positive slopes obtained from the regression equations of the datasets generated through bootstrap resampling (100 iterations).

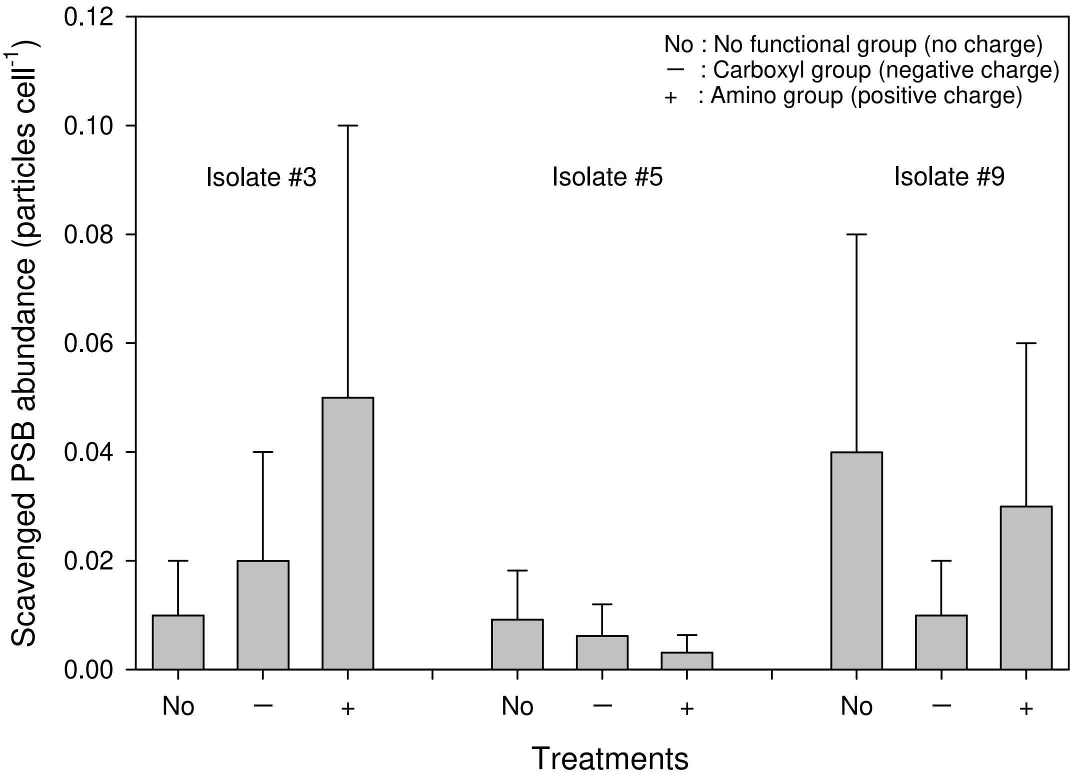

Fig. S11. Abundance of scavenged nanoparticles from polystyrene beads (PSBs) with different surface charges by three bacterial isolates. Values represent the mean ± standard deviation ($n = 5$). The total number of analyzed cells ranged from 237 to 866 for each treatment. There were no significant differences among PSBs with different surface charges for each isolate (p >0.05, one-way ANOVA).

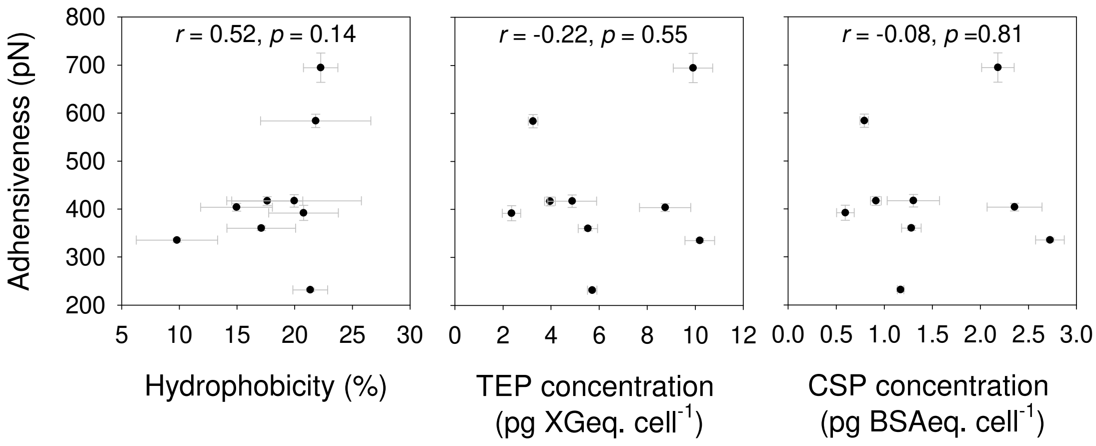

Fig. S12. Relationships between adhesiveness and chemical properties of bacterial cell surfaces. Each plot is the mean ± standard errors (number of analyzed cells = 38–103 for adhesiveness, and n = 9 and n = 3 for hydrophobicity and concentrations of extracellular polymeric substances (TEP: transparent exopolymer particles; CSP: Coomassie stained particles), respectively). Values of adhesiveness and chemical properties of each isolate were shown in Supporting Information Tables S2 and S4, respectively. Spearman's rank correlation (r) and statistical significance (p) are given for each relationship.

References

Cerf, A., J. C. Cau, C. Vieu, and E. Dague. 2009. Nanomechanical properties of dead or alive single-patterned bacteria. Langmuir **25:** 5731–5736.

Chen, Y.Y., C.C. Wu, J.L. Hsu, H.L. Peng, H.Y. Chang, and T.R. Yew. 2009. Surface rigidity change of *Escherichia coli* after filamentous bacteriophage infection. Langmuir. **25:** 4607–4614. doi: 10.1021/la8036346.

Cisternas-Novoa, C., C. Lee, and A. Engel. 2014. A semi-quantitative spectrophotometric, dye-binding assay for determination of Coomassie Blue stainable particles. Limnol. Oceanogr. Methods **12:** 604–616.

Eaton, P., J.C. Fernandes, E. Pereira, M.E. Pintado, and F.X. Malcata. 2008. Atomic force microscopy study of the antibacterial effects of chitosans on Escherichia coli and Staphylococcus aureus. Ultramicroscopy **108:** 1128–1134. doi: 10.1016/j.ultramic.2008.04.015.

Francius, G., O. Domenech, M.P. Mingeot-Leclercq, and Y.F. Dufrene. 2008. Direct observation of *Staphylococcus aureus* cell wall digestion by lysostaphin. J Bacteriol. **190:** 7904–7909. doi: 10.1128/JB.01116-08.

Gaboriaud, F., B.S. Parcha, M.L. Gee, J.A. Holden, and R.A. Strugnell. 2008. Spatially resolved force spectroscopy of bacterial surfaces using force-volume imaging. Colloid Surface B **62:** 206–213. doi: 10.1016/j.colsurfb.2007.10.004.

Harimawan, A., A. Rajasekar, and Y. P. Ting. 2011. Bacteria attachment to surfaces -AFM force spectroscopy and physicochemical analyses. J. Colloid Interface Sci. **364:** 213–218.

Kumar, U., K. Vivekanand, and P. Poddar. 2009. Real-time nanomechanical and topographical mapping on live bacterial cells – *Brevibacterium casei* under stress due to their exposure to Co^{2+} ions during microbial synthesis of Co_3O_4 nanoparticles. J. Phys. Chem. B. **113:** 7927–7933. doi: 10.1021/jp902698n.

Lemke, M. J., P. F. Churchill, and R. G. Wetzel. 1995. Effect of substrate and cell surface hydrophobicity on phosphate utilization in bacteria. Appl. Environ. Microbiol. **61:** 913–919.

Liu, Y., C. H. Yang, and J. Li. 2007. Influence of extracellular polymeric substances on *Pseudomonas aeruginosa* transport and deposition profiles in porous media. Environ. Sci. Technol. **41:** 198−205

Passow, U., and A. L. Alldredge. 1995. A dye-binding assay for the spectrophotometric measurement of transparent exopolymer particles (TEP). Limnol. Oceanogr. **40:** 1326–1335.

Pelling, A. E., Y. Li, W. Shi, and J. K. Gimzewski. 2005. Nanoscale visualization and characterization of *Myxococcus xanthus* cells with atomic force microscopy. Proc. Natl. Acad. Sci. USA 102: 6484–6489.

Rosenberg, M., D. Gutnick, and E. Rosenberg. 1980. Adherence of bacteria to hydrocarbons: A simple method for measuring cell-surface hydrophobicity. FEMS Microbiol. Lett. **9:** 29–33.

Thio, B.J., and J.C. Meredith. 2008. Quantification of *E. coli* adhesion to polyamides and polystyrene with atomic force microscopy. Colloids Surf. B Biointerfaces **65(2):** 308–312. doi: 10.1016/j.colsurfb.2008.05.005.

van der Aa, B. C., and Y. F. Dufrêne. 2002. In situ characterization of bacterial extracellular polymeric substances by AFM. Colloid Surf. B **23:** 173–182.

Vandevivere, P., and D. L. Kirchman. 1993. Attachment stimulates exopolysaccharide synthesis by a bacterium. Appl. Environ. Microbiol. **59:** 3280–3286.

Zhang, W., A.G. Stack, and Y. Chen. 2011. Interaction force measurement between *E. coli* cells and nanoparticles immobilized surfaces by using AFM. Colloids Surf. B Biointerfaces **82(2):** 316–324, doi: 10.1016/j.colsurfb.2010.09.003.
